# Cx43-Gap Junctions Accumulate at the Cytotoxic Immunological Synapse Enabling Cytotoxic T Lymphocyte Melanoma Cell Killing

**DOI:** 10.3390/ijms20184509

**Published:** 2019-09-12

**Authors:** Francisca Hofmann, Mariela Navarrete, Javiera Álvarez, Israel Guerrero, María Alejandra Gleisner, Andrés Tittarelli, Flavio Salazar-Onfray

**Affiliations:** 1 Disciplinary Program of Immunology, Institute of Biomedical Sciences, Faculty of Medicine, University of Chile, 8380453 Santiago, Chile; fcah.vega@gmail.com (F.H.); mariela.navarrete.s@gmail.com (M.N.); jalvarezm1011@gmail.com (J.Á.); israel.guerrero.l@gmail.com (I.G.); alejandra.gleisner@gmail.com (M.A.G.); 2 Millennium Institute on Immunology and Immunotherapy, Faculty of Medicine, University of Chile, 8380453 Santiago, Chile

**Keywords:** connexin 43, gap junctions, melanoma, cytotoxic T lymphocyte, cytotoxic immunological synapse

## Abstract

Upon tumor antigen recognition, cytotoxic T lymphocytes (CTLs) and target cells form specialized supramolecular structures, called cytotoxic immunological synapses, which are required for polarized delivery of cytotoxic granules. In previous reports, we described the accumulation of connexin 43 (Cx43)-formed gap junctions (GJs) at natural killer (NK) cell–tumor cell cytotoxic immunological synapse. In this report, we demonstrate the functional role of Cx43-GJs at the cytotoxic immunological synapse established between CTLs and melanoma cells during cytotoxicity. Using confocal microscopy, we evaluated Cx43 polarization to the contact site between CTLs isolated from pMEL-1 mice and B16F10 melanoma cells. We knocked down Cx43 expression in B16F10 cells and evaluated its role in the formation of functional GJs and the cytotoxic activity of CTLs, by calcein transfer and granzyme B activity assays, respectively. We found that Cx43 localizes at CTL/B16F10 intercellular contact sites via an antigen-dependent process. We also found that pMEL-1 CTLs but not wild-type naïve CD8^+^ T cells established functional GJs with B16F10 cells. Interestingly, we observed that Cx43-GJs were required for an efficient granzyme B activity in target B16F10 cells. Using an HLA-A2-restricted/MART-1-specific CD8^+^ T-cell clone, we confirmed these observations in human cells. Our results suggest that Cx43-channels are relevant components of cytotoxic immunological synapses and potentiate CTL-mediated tumor cell killing.

## 1. Introduction

Gap junctions (GJs) are clusters of intercellular channels found at the plasma membrane that allow direct communication between the cytoplasm of adjacent cells. Each GJ channel is formed by two hexameric hemichannels, also known as connexons, one provided by each of the two contacting cells. In turn, each hemichannel is composed of six transmembrane proteins called connexins (Cx) [1]. Once functional GJs are established, these channels allow the exchange of small molecules (up to ≈1.4 nm) between the cytoplasm of adjacent cells, including adenosine triphosphate (ATP), cyclic adenosine monophosphate (cAMP), cyclic guanosine monophosphate-adenosine monophosphate (cGAMP), inositol triphosphate (IP_3_), Ca^2+^, glutamate, microRNAs, and small peptides [2]. The Cx gene family is composed of 21 different members in humans and 20 in mice, where different isoforms are expressed in a tissue-specific manner and determine channel properties. Cx43 is expressed almost ubiquitously and it is the main Cx member expressed in the immune system cells [3,4,5].

Different reports describe the participation of GJs as modulators of key immunological processes, including anti-tumor responses [6]. For instance, it has been reported that GJs are involved in immunoglobulin secretion and cytokine production [7], Treg cell-mediated immune suppression via delivery of cAMP [8], and in the transfer and cross-presentation of antigenic peptides [9], including melanoma-associated antigens (MAAs) [10]. This antigenic peptide transfer mechanism has also been observed between gut-resident antigen-presenting cells (APCs) [11] and, in addition, it was suggested that the Cx43-mediated transfer of antigenic peptides from melanoma cells to dendritic cells (DCs) could be the major mechanism of tumor-associated antigen cross-presentation occurring in vivo, leading to the activation of tumor antigen-specific cytotoxic T lymphocytes (CTLs) and the subsequent elimination of the tumor [12,13]. Furthermore, recent evidence suggests that Cx43 is a component of the immunological synapses, and that Cx43-mediated GJ intercellular communications (Cx43-GJICs) are required for DC activation [14] and antigen-dependent DC-mediated CD4^+^ T-cell activation [15]. At the immunological synapse, GJs formed by Cx43 (Cx43-GJs) mediate the crosstalk between DCs and T cells in an antigen-dependent manner, observing this phenomenon in both murine and human cells [15,16]. Similarly, we reported that Cx43 channels accumulate at the immunological synapse established between mature DCs and resting natural killer (NK) cells, allowing the bidirectional communication between these cells. The blockade of Cx43-GJs leads to a strong decrease in the DC-mediated NK-cell activation, measured by the expression of the activation markers CD69 and CD25, and to a reduced IFN-γ secretion [17]. Moreover, Cx43 localizes in the cytotoxic immunological synapses established between NK cells and target tumor cells and mediates intercellular communications that participate in NK cell-mediated cytotoxicity [17,18]. Cx43-GJIC does not affect tumor-induced NK-cell degranulation but regulates an efficient Ca^2+^ influx into the target tumor cells, which contributes to granzyme B (GrzmB) activity inside target tumor cells, leading to cell apoptosis [17,18]. Despite the fact that cytotoxic immunological synapses of CTLs and NK cells have some functional and structural similarities [19], the participation of Cx43-GJs in the CTL cytotoxic activity remains unexplored. In this report, we aimed to evaluate the involvement of Cx43-GJs at the cytotoxic immunological synapse and the cytotoxic capacity of CTLs using the melanoma-specific pMEL-1 murine model [20] and the B16F10 melanoma cell line, as well as a human HLA-A2-restricted/MART-1-specific CTL clone (CdL43-1) [10] and a HLA-A2^+^ MART-1^+^ human melanoma cell line (Mel1) as effector and target tumor cells, respectively. Here, we describe for the first time that Cx43 localizes at the contact site of CTL/target melanoma cell conjugates, allowing the antigen-driven GJ-mediated intercellular communication between these cells during the CTL cytotoxic immunological synapse establishment. The knocking down of Cx43 or the inhibition of Cx43 channels using the Cx43 mimetic peptide gap27 leads to a decrease in this intercellular communication and, subsequently, to the GrzmB activity in the melanoma target cells, suggesting that Cx43-GJ formation is necessary for an efficient CTL-mediated tumor cell killing.

## 2. Results

### 2.1. Cx43 Polarizes to the pMEL-1 CTL-B16F10 Melanoma Cell Contact Site Upon Cytotoxic Immunological Synapse Formation

**Figure 1:**
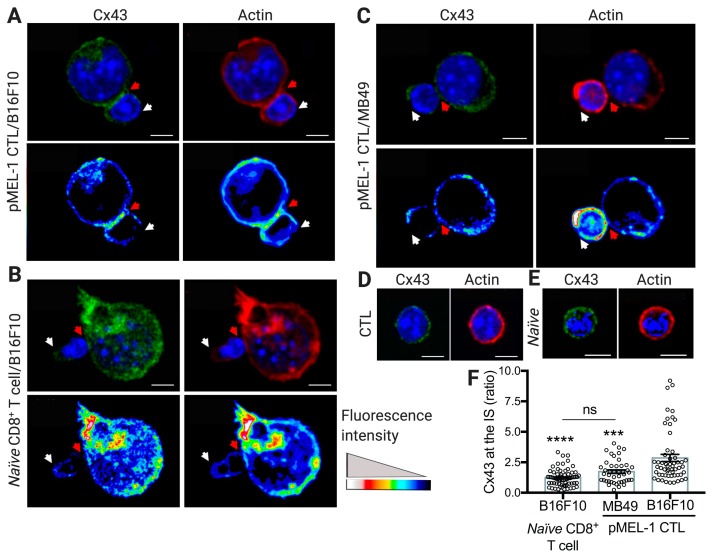
Connexin-43 (Cx43) accumulates at the pMEL-1 cytotoxic T lymphocyte (CTL)/B16F10 melanoma cell contact site upon cytotoxic immunological synapse (IS) formation. Representative images for cell conjugates formed after 30 min of co-culture of pMEL-1 CTLs or naïve CD8^+^ T cells with wheat germ agglutinin (WGA) pre-loaded (not shown) target (**A**,**B**) B16F10 cells or (**C**) MB49 cells. The distributions of both Cx43 (green) and F-actin (rhodamine-phalloidin, red) at the cytotoxic IS are indicated by red arrows and were analyzed by immunofluorescence and confocal microscopy. (**A**–**E**) Hoechst nuclear staining is shown in blue. Representative images of Cx43 and actin staining in (**D**) unconjugated pMEL-1 CTL and (**E**) naïve CD8^+^ T cell. (**A**–**C**) Pseudocolor images for the distribution of Cx43 and F-actin are shown at the bottom of each figure. (**A**–**E**) Scale bars = 5 μm. (**F**) Quantification of Cx43 accumulation at the cytotoxic IS as ratio of Cx43 fluorescence intensity in the cell-cell contact site (red arrows) versus the opposite site (white arrows). *** *p* < 0.001, **** *p* < 0.0001, versus CTL/B16F10 conjugates; ns, non-significant (one-way ANOVA, Tukey’s multiple comparison test); *n* = approximately 60 cell conjugates by condition, two independent experiments.

Previously, we and others have demonstrated that Cx43 channels accumulate at the immunological synapses during DC-T cell and DC-NK cell interactions [15,16,17] and at the NK cell-tumor cell cytotoxic immunological synapse [17,18], allowing DC-mediated T- and NK-cell activation and NK cell-mediated target tumor cell killing, respectively. Given that the cytotoxic immunological synapses formed by NK cells and CTLs share some structural features [19], in this report we aimed to examine whether Cx43 channels have a role in CTL-target tumor cell immunological synapses. CTL cytotoxic immunological synapses occur when the T-cell receptor (TCR) recognizes its cognate antigenic peptide associated with major histocompatibility complex (MHC) class I molecules [19]. In order to test the role of Cx43 in functionally active cytotoxic immunological synapses, we used the murine model pMEL-1 (C57BL6 genetic background), which is characterized by having CD8^+^ T cells that express a transgenic Vβ13 chain in its TCR that specifically recognizes the peptide gp100_25–33_ (XXXRNQDWL) of the gp100 protein bound to H-2 D^b^ molecules. The gp100 protein is considered an MAA, and it is expressed in melanocytic lineage cells and overexpressed in different melanoma cell lines, including the syngeneic B16F10 melanoma cells [20,21]. First, we differentiated pMEL-1 CTLs in vitro from pMEL-1 mice splenocytes by incubating them with interleukin (IL)-2 and the hgp100_25–33_ peptide for six days. This procedure allowed the generation of fully differentiated pMEL-1 effector CTLs, characterized by the high expression of activation and memory markers CD25 and CD44 on the CD8^+^Vβ13^+^ T-cell population (Figure S1A, Supplementary Materials). The cellular distribution of Cx43 was then assessed by confocal microscopy in pMEL-1 CTL/B16F10 cell conjugates. Cx43 was found to preferentially accumulate in the interface between pMEL-1 CTLs and B16F10 cells, which, as indicated by the accumulation of F-actin [19], would correspond to stable cytotoxic immunological synapses (Figure 1A). When naïve CD8^+^ T cells isolated from splenocytes of wild-type C57BL6 mice (Figure S1B, Supplementary Materials) were co-cultured with B16F10 cells, functional cytotoxic immunological synapses were not formed, as expected, and therefore Cx43 channels are not observed at the cell-to-cell interface (Figure 1B). In addition, Cx43 was not detected in the cell interface of cell conjugates of pMEL-1 CTLs and syngeneic bladder carcinoma MB49 cells, which do not express the MAA gp100 and therefore do not trigger the formation of functional cytotoxic immunological synapses with gp100_25–33_-specific CD8^+^ T cells (Figure 1C). Of note, the distribution of Cx43 and F-actin was observed uniformly expressed on the cell surface in unconjugated pMEL-1 CTLs or naïve CD8^+^ T cells (Figure 1D,E), and both kinds of T cells showed similar Cx43 expression levels (Figure 1D,E and Figure S1C, Supplementary Materials). These results indicate that Cx43 polarizes to the cytotoxic immunological synapses of murine CTLs and target tumor cells in an antigen-dependent manner (Figure 1F).

### 2.2. pMEL-1 CTLs Form Functional Cx43-GJ-Mediated Intercellular Communications with B16F10 Melanoma Cells

**Figure 2:**
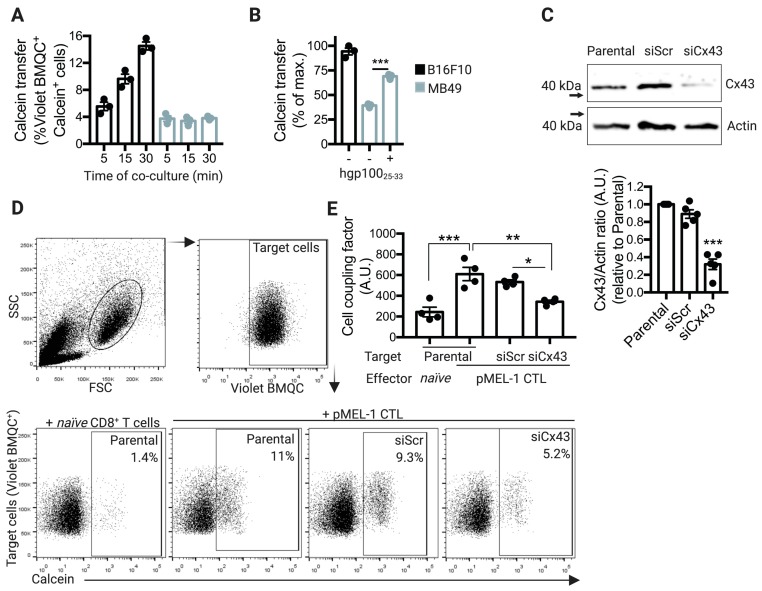
pMEL-1 cytotoxic T lymphocytes (CTLs) form functional connexin-43 (Cx43)-mediated gap junction (GJ) communications with B16F10 melanoma cells. (**A**) B16F10 or MB49 cells were pre-loaded with the CellTracker Violet BMQC and co-cultured for different time points (as indicated) with calcein-AM pre-loaded pMEL-1 CTLs, at a 1:5 ratio. Calcein transfer from effector to target tumor cells was assessed by flow cytometry. The bar graph shows Violet BMQC^+^calcein^+^ cells. (**B**) Calcein transfer from pMEL-1 CTLs to target tumor cells was evaluated as described before, after 30 min of co-culture. MB49 cells were pre-loaded or not with hgp100_25–33_ peptide before co-culturing with pMEL-1 CTLs. The bar graph shows Violet BMQC^+^calcein^+^ cells as a percentage of the maximum calcein transfer. (**C**) B16F10 cells were transfected with siRNAs against Cx43 (siCx43) or control-scrambled siRNAs (siScr). The expression of Cx43 and actin was assessed three days after transfection by Western blot in transfected or non-transfected (parental) B16F10 cells, and Cx43/actin ratios were quantified by ImageJ software. The bar graph at the bottom shows the average of Cx43 expression depicted as Cx43/actin ratio relative to parental untransfected cells (*n* = 5 independent experiments). (**D**) Representative dot plots showing the strategy for Cx43-GJ communication measuring. Target (parental, siScr- or siCx43-transfected B16F10) cells were pre-loaded with the CellTracker Violet BMQC and co-cultured for 30 min with calcein-AM pre-loaded effector cells (naïve CD8^+^ T cells or pMEL-1 CTLs), at a 1:5 ratio. Calcein transfer from effector to target tumor cells was assessed by flow cytometry. The numbers in the dot plots represent the percentage of Violet BMQC^+^calcein^+^ cells. (**E**) The bar graph shows the cell coupling factor calculated as (%Violet BMQC^+^calcein^+^ cells × Calcein MFI of Calcein^+^ cells)/100. * *p* < 0.05; ** *p* < 0.01; *** *p* < 0.001; ns = non-significant (one-way ANOVA, Tukey’s multiple comparison test). A.U., arbitrary units.

To determine whether pMEL-1 CTLs and target B16F10 melanoma cells can communicate with each other through Cx43 channels upon cytotoxic immunological synapse formation, we performed calcein transfer assays by flow cytometry analysis, as described before [17]. In contrast to MB49 cells, which did not induce Cx43 polarization to the contact site with pMEL-1 CTLs, B16F10 melanoma cells did acquire calcein from pMEL-1 CTLs after 30 min of co-culture (Figure 2A), concomitant with the Cx43 polarization to the cell-to-cell contact site. When we forced the recognition of MB49 cells by pMEL-1 CTLs through the pre-incubation of target tumor cells with the antigenic peptide hgp100_25–33_, CTLs effectively transferred calcein to the MB49 tumor cells (Figure 2B), indicating that the cell coupling between CTLs and target tumor cells is an antigen-dependent process. In order to test if the cell coupling between pMEL-1 CTLs and B16F10 cells is a Cx43-dependent mechanism, we knocked down the expression of Cx43 in B16F10 melanoma cells using specific anti-Cx43 siRNAs (siCx43). Our results showed that the knocking-down efficiency of Cx43 in these cells was approximately 70%, as compared with Cx43 expression observed in parental (non-transfected B16F10 cells) or B16F10 cells transfected with control-scrambled siRNAs (siScr) (Figure 2C). In concordance with the localization of Cx43 at the intercellular contact site, pMEL-1 CTLs but not wild-type naïve CD8^+^ T cells transferred calcein to B16F10 parental cells, and this cell coupling was partially but significatively decreased when Cx43 was silenced in the target tumor cells (Figure 2D,E). Overall, our results suggest that upon CTL cytotoxic immunological synapse establishment, Cx43 polarizes to the synapse allowing the effector/target cell coupling via Cx43-GJ channels.

### 2.3. Cx43 is Required for GrzmB-Mediated Cytotoxicity of pMEL-1 CTLs against B16F10 Melanoma Cells 

**Figure 3:**
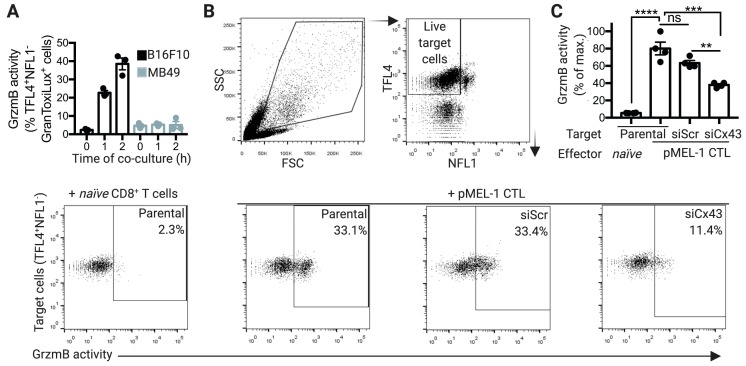
Connexin-43 gap junctions (Cx43-GJs) are required for granzyme b (GrzmB)-mediated cytotoxicity of pMEL-1 cytotoxic T lymphocytes (CTLs) against B16F10 melanoma cells. (**A**) B16F10 or MB49 cells were pre-stained with TFL4 (CellTracker) and NFL1 (viability marker) and co-cultured for different times (as indicated) with pMEL-1 CTLs, at a 1:5 ratio, in the presence of a permeable fluorogenic substrate for GrzmB (GranToxiLux; GrzmB activity). GrzmB activity was evaluated on TFL4^+^NFL1^−^ target tumor cells by flow cytometry. (**B**) Representative dot plots showing the strategy for measuring GrzmB activity in target tumor cells co-cultured with effector cells. Target cells (parental, siScr- or siCx43-transfected B16F10) were pre-stained with TFL4 and NFL1 and co-cultured for 2 h with effector cells (naïve CD8^+^ T cells or pMEL-1 CTLs), at a 1:5 ratio, in the presence of GranToxiLux substrate. The numbers in the dot plots represent the percentage of TFL4^+^NFL1^−^GranToxiLux^+^ cells. (**C**) The bar graph shows the GrzmB activity in target tumor cells (TFL4^+^NFL1^−^GranToxiLux^+^) as a percentage of the maximum (target tumor cell: parental/effector cell: pMEL-1 CTL); *n* = 4 independent experiments. ** *p* < 0.01; *** *p* < 0.001; **** *p* < 0.0001; ns = non-significant (one-way ANOVA, Tukey’s multiple comparison test).

Cx43-GJ channels have been described as mediators of intercellular communications between NK cells and target tumor cells during NK-cell cytotoxic immunological synapse formation. Recent reports have shown that Cx43-channel inhibition during NK-cell immunological synapse establishment decreases GrzmB activity in target tumor cells and NK-cell anti-tumor cytotoxic capacity without affecting the degranulation process [17,18]. As expected, we detected significant levels of GrzmB activity in B16F10 cells, but not in MB49 cells, after 1 or 2 h of co-culture with pMEL-1 CTLs (Figure 3A). Similarly to the results of Cx43 localization at the cytotoxic immunological synapse and the Cx43-mediated intercellular communication, pMEL-1 CTLs but not wild-type naïve CD8^+^ T cells were able to induce GrzmB activity on B16F10 parental cells, which was significatively decreased when Cx43 was silenced in the target tumor cells (Figure 3B,C).

### 2.4. Cx43-GJ Intercellular Communications Are Required for Optimal GrzmB-Mediated Cytotoxicity of Human CTLs against Melanoma Cells

**Figure 4:**
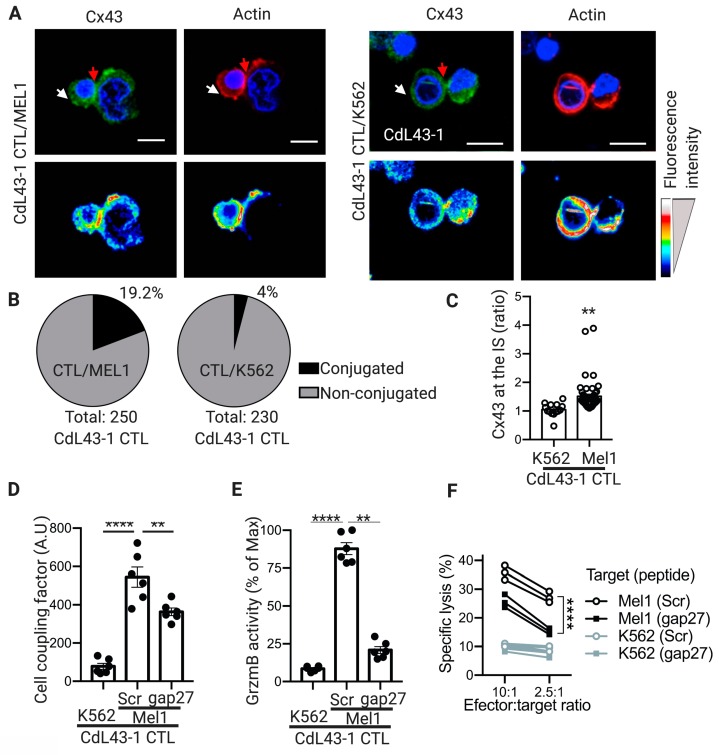
Connexin-43 (Cx43) localizes at cytotoxic immunological synapses (IS) and contributes to cell coupling and granzyme b (GrzmB)-mediated cytotoxicity of human cytotoxic T lymphocytes (CTLs) against melanoma cells. (**A**) Representative images for cell conjugates formed after 30 min of co-culture of HLA-A2-restricted/MART-1-specific CD8^+^ T-cell clone (CdL43-1 CTL) with wheat germ agglutinin (WGA) pre-loaded (not shown) target Mel1 cells (left) or K562 cells (right, negative control). The distributions of both Cx43 (green) and F-actin (rhodamine-phalloidin, red) at the cytotoxic IS are indicated by red arrows and were analyzed by immunofluorescence and confocal microscopy. Hoechst nuclear staining is shown in blue. Pseudocolor images of the distribution for Cx43 and F-actin are shown at the bottom of each figure. Scale bars = 5 μm. (**B**) Quantification of the percentage of CdL43-1 CTLs conjugated to target tumor cells after co-culture with Mel1 (left) or K562 (right) cells. (**C**) Quantification of Cx43 accumulation at the cytotoxic IS as ratio of Cx43 fluorescence intensity in the cell-cell contact site (red arrows) versus at the opposite site (white arrows). (**D**) Target (K562 or Mel1) tumor cells were pre-loaded with the CellTracker Violet BMQC and co-cultured for 1 hour with calcein-AM pre-loaded CdL43-1 CTL effector cells at 1:3 ratios, in the presence of Cx43 mimetic inhibitory (gap27) or control (Scr) peptides (300 μM). Calcein transfer from effector to target tumor cells was assessed by flow cytometry. The bar graph shows the cell coupling factor calculated as (%Violet BMQC^+^calcein^+^ cells × Calcein MFI of Calcein^+^ cells)/100. (**E**) Mel1 or K562 cells were pre-stained with TFL4 (CellTracker) and NFL1 (viability marker) and co-cultured for 2 h with CdL43- CTLs, at a 1:3 ratio, in the presence of a permeable fluorogenic substrate for GrzmB (GranToxiLux; GrzmB activity), and gap27 or Scr peptides. GrzmB activity was evaluated on TFL4^+^NFL1^−^ target tumor cells by flow cytometry. The bar graph shows the GrzmB activity in target tumor cells (TFL4^+^NFL1^−^GranToxiLux^+^) as a percentage of the maximum (target tumor cell: Mel1; peptide: Scr). (**F**) CdL43-1 CTLs were co-cultured with Mel1 or K562 cells at different effector:target cell ratios in the presence of gap27 or Scr peptides. Cytotoxicity was assessed by conventional ^51^Cr release assays. The results are plotted as a percentage of specific lysis. All the results are from three independent experiments. ** *p* < 0.01; **** *p* < 0.0001 (one-way ANOVA, Tukey’s multiple comparison test, or two-tailed Student’s *t*-test (C)).

The contribution of Cx43 channels on the CTL cytotoxic immunological synapse was further investigated using a human cell model. As effector human CTLs, we used an HLA-A2-restricted/MART-1-specific CD8^+^ T-cell clone (CdL43-1), obtained from a melanoma patient [10]. As positive or negative target tumor cells, we used an HLA-A2^+^/MART-1^+^ human melanoma cell line (Mel1) and a myelogenous leukemia cell line K562 (HLA^−^), respectively. We found that, as with murine CTLs, Cx43 polarizes to the cell-cell contact zone of CdL43-1 CTL/Mel1 conjugates, whereas it distributes homogeneously in CdL43-1 CTL/K562 conjugates. This was associated with effective cytotoxic immunological synapse formations, as indicated by F-actin remodeling (Figure 4A–C). As expected, CdL43-1 CTL did not form stable conjugates with the HLA^−^ K562 cells, as indicated by the low percentage of cell conjugates observed under microscopy (Figure 4B). The polarization of Cx43 at the cytotoxic immunological synapse of human CTLs also correlates with increased Cx43-mediated cell coupling with target melanoma cells, as indicated by calcein transfer assays and the inhibition of Cx43 channels by the Cx43-mimetic peptide gap27 (Figure 4D). Similarly to the results of Cx43 localization at the cytotoxic immunological synapse and Cx43-mediated intercellular communication, CdL43-1 CTLs were able to induce GrzmB activity and target tumor cell killing on Mel1 cells but not in K562 cells, which was significatively decreased when Cx43 channels were blocked with gap27 mimetic peptide (Figure 4E,F).

## 3. Discussion

The establishment of anti-tumor immune responses requires strongly coordinated and regulated communications between immune cells and between immune cells and target tumor cells. In this sense, both DC-T cell synapses formed during DC-mediated T-cell activation and CTL or NK cell/target tumor cell synapses, established during the former’s effector phases, are supramolecular structures that regulate fundamental intercellular communication mechanisms required for an effective control of tumor cells by the immune system. The mechanisms allowing the establishment of both types of immunological synapses share some molecular features, including the rearrangement of the cytoskeleton, receptors, and adhesion molecules in the intercellular contact zone. In the cytotoxic immunological synapses, those molecular rearrangements ensure the polarized release of perforin- and granzyme-containing cytotoxic granules towards the target cells, which finally triggers apoptosis [19,22,23]. Previously, the participation of Cx43 channels as functional structures of immunological synapses formed among DCs and CD4^+^ T cells, between DCs and NK cells, and among NK cells and target tumor cells has been described [15,16,17,18]. These channels were shown to be necessary for DC-mediated activation of both CD4^+^ T cells and NK cells, as well as for the efficient NK cell-mediated tumor cell killing. Taking into account the fact that the immunological synapses formed by CTLs and NK cells have functional and structural similarities [19,24,25], we aimed to determine in this report whether Cx43-GJ channels are also involved in the CTL/target tumor cell cytotoxic immunological synapses. To this end, we used CTLs isolated from pMEL-1 mice, which specifically recognize the epitope XXXRNQDWL for the MAA gp100 endogenously expressed by the syngeneic B16F10 melanoma cells, and a human CTL clone specific for the HLA-A2-restricted MART-1_27–35_ antigen, an MAA expressed in the HLA-A2^+^ Mel1 melanoma cell line.

Upon antigen recognition via TCR/cognate antigenic peptide-MHC I complex (pMHC I) interactions, a rearrangement of the actin cytoskeleton occurs at the proximity of the plasma membrane of T cells, leading to the formation of an actin ring structure along the site of the intercellular contact, which is indicative of an efficient cytotoxic immunological synapse formation [19]. We observed a structure resembling actin rings in pMEL-1 CD8^+^ CTLs upon antigen recognition of target B16F10 melanoma cells (Figure 1) and, only upon this antigen-specific driven process, Cx43 polarizes to the cell-to-cell contact sites, indicating that Cx43 accumulates in the cytotoxic immunological synapses of CTLs. It is known that once Cx proteins are translated, they indirectly bind to the cytoskeleton through interactions with motor proteins, such as myosin in the case of the actin cytoskeleton and kinesins in the case of the microtubule cytoskeleton. Through these interactions, Cxs traffic towards the cell plasma membrane for the formation of hemichannels and GJs [26]. In the present report, we observed that the polarization of Cx43 to the cell-to-cell contact site is concomitant with the F-actin accumulation, exclusively in the antigen-specific condition, suggesting that Cx43 could be mobilized towards the cytotoxic immunological synapses through actin filaments. However, this point needs to be further addressed. Additionally, a previous report indicates that the recruitment of T cell Cx43 to the contact area of CD3- and CD28-coated beads is dependent on the actin cytoskeleton but not on microtubules [15]. Furthermore, it has been reported that Cx43 interacts with the zonula occluden (ZO) proteins ZO-1 and ZO-2 [27]. The interaction of Cx43 with ZO-1 regulates the rate of undocked connexon aggregation into GJs [28]. In addition, ZO-1 and ZO-2 bind to the actin cytoskeleton in the cellular plasma membrane to form tight junctions [29,30] and the participation of ZO-2 in the immunological synapse established among T cells and APCs has recently been described [31]. These data allow us to suggest that Cx43 may interact with ZO-1 and/or ZO-2 for being located in the actin rings at the distal supramolecular activation cluster (dSMAC) of CTL cytotoxic immunological synapses. Of note, we previously demonstrated that Cx43 accumulates preferentially in the dSMAC of immunological synapses established between T cells and DCs [15].

**Figure 5:**
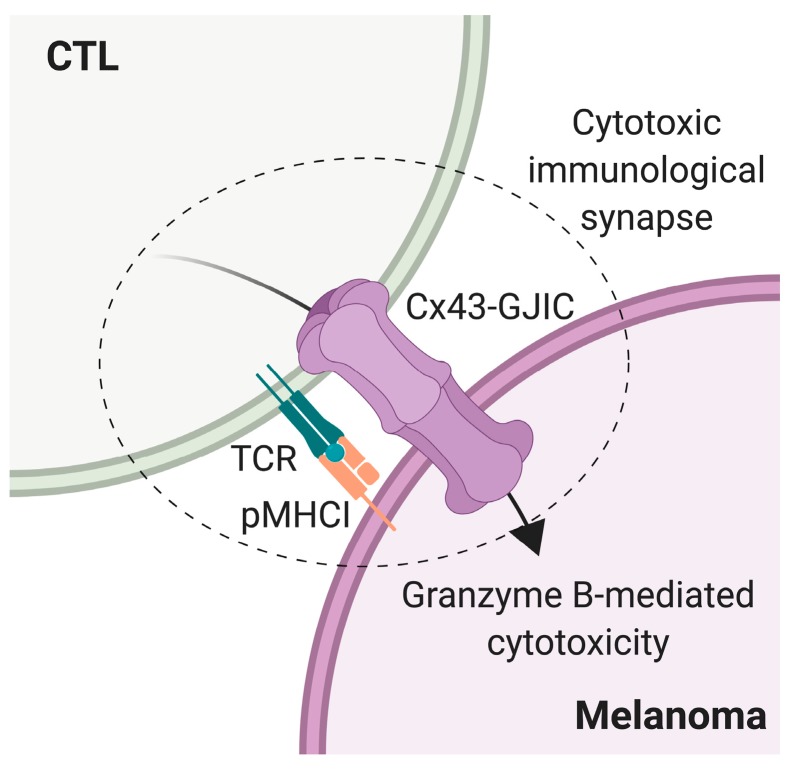
Model of connexin-43 (Cx43)-gap junction (GJ) intercellular communication (Cx43-GJIC) in the cytotoxic T lymphocyte (CTL)-mediated melanoma cell killing. Upon cytotoxic immunological synapse formation, initiated by T cell receptor (TCR)-cognate antigenic peptide-MHC I (pMHCI) interaction, Cx43-GJ channels polarize to the CTL-melanoma cell contact site, allowing Cx43-mediated cell coupling and the efficient activity of CTL-derived granzyme B on the target tumor cell.

Our results indicate that the antigen-driven polarization of Cx43 at the CTL cytotoxic immunological synapse is associated with an increase in CTL/target tumor cell coupling, measured as Cx43-dependent calcein transfer (Figure 2). Impairment on Cx43-mediated cell coupling, generated by Cx43 knockdown in B16F10 cells, was associated with diminished GrzmB activity in the target tumor cells (Figure 3). This suggests that through a yet uncharacterized mechanism, Cx43-mediated intercellular communications between CTLs and target tumor cells are necessary for an efficient cell death-leading GrzmB activity in target tumor cells, in both murine and human cells (Figure 4 and Figure 5). We speculate that two possible complementary mechanisms may explain the role of Cx43 channels in the activity of CTL cytotoxic immunological synapses. (i) Cx43 channels could act as a form of intercellular adhesion along with other adhesion molecules present in the cytotoxic immunological synapses, as LFA-1 integrin-mediated binding to ICAM-1 on target cells [32]. In this context, it has been reported that Cx43 regulates cell adhesion in neurons, glioma cells, B cells, and monocytes [33,34,35]. More recently, it has been observed that chimeric antigen receptor T (CAR-T) cells form potent cytotoxic immunological synapses less reliant on LFA-1 to form stable conjugates than TCR immune synapses [36]. Interestingly, the authors observed that CAR-T cell synapses, whose signaling was stronger and more rapid than in TCR immunological synapses, were more enriched in Cx43, suggesting that Cx43 could be essential for the formation of effective cytotoxic immunological synapses even in the presence of disorganized LFA-1 adhesion rings [36]. (ii) Cx43 channels could allow the transfer of small signaling molecules from CTLs to target tumor cells, which contribute finally to efficient GrzmB activity in the former cell. A signaling molecule that fulfills this requirement is Ca^2+^, whose influx in target cells was reported as necessary for NK cell- and CTL-mediated GrzmB-induced apoptosis in target cells [37,38,39]. In those reports, it has been shown that perforin and GrzmB are endocytosed in a Ca^2+^-dependent manner in large endosomes, called gigantosomes, within the target cell near the synapse. The authors proposed that the disruption of this gigantosome leads to the release of GrzmB into the cytosol. Therefore, a rise of intracellular Ca^2+^ concentrations in target cells is required for the efficient CTL and NK cell killing of their targets. It has been proven that pores formed by perforin allow this Ca^2+^ influx in target cells [37,38,39]. In addition, we have previously shown that the inhibition of Cx43 channels in NK cell/K562 conjugates decreases NK cell-mediated Ca^2+^ influx in the target tumor cells [17]. These data suggest that Cx43 channels could collaborate with perforin pores in inducing a Ca^2+^ influx and thus play a role in the gigantosome formation and/or contribute to their content release, leading to an efficient GrzmB activity in tumor cells killed by CTLs. Further studies are needed to conclusively demonstrate this hypothesis.

This is the first study, to our knowledge, demonstrating that Cx43-GJ formation is important for CTL-mediated target tumor cell lysis. Our data indicate that reduced Cx43 expression may be a valuable mechanism for the immune evasion of malignant or pathogen-infected cells. Indeed, it is known that many tumor cells downregulate Cx43 expression [6]. Therefore, examining the expression levels of Cx43 in tumors may be an important strategy to design appropriate immune therapeutic treatments dependent on CTL activity.

## 4. Materials and Methods

### 4.1. Mice

Wild-type C57BL6 and transgenic pMEL-1 (C57BL6 background) mice [20] were bred at Universidad de Chile. For all experiments, mice between 8 and 12 weeks of age were bred in specific pathogen-free conditions. All animal experiments were performed in accordance with institutional guidelines for animal care and were approved by the Ethical Review Committees at the Faculty of Medicine of the Universidad de Chile, ethical number: CBA FMUCH 0825 (approval date: 2 October 2015).

### 4.2. Cell Lines

B16F10 melanoma (ATCC^®^ CRL-6475™), MB49 urothelial carcinoma (RRID:CVCL-7076), K562 (ATCC^®^ CCL-243™), and Mel1 [17] cell lines were maintained at 37 °C under 5% CO_2_ and 95% relative humidity in RPMI-1640 medium supplemented with 10% fetal bovine serum (FBS) and 1% streptomycin/penicillin (all from Corning™, Manassas, VA, USA).

### 4.3. pMEL-1 CTL Differentiation and Naïve CD8^+^ T-Cell Purification

To differentiate pMEL-1 CTLs, total splenocytes of pMEL-1 mice (8 to 12 weeks old) were obtained by perfusion of spleens and then cultured for six days in RPMI GlutaMAX™ culture medium supplemented with 10% FBS (HyClone^TM^, South Logan, Utah, USA), 1% streptomycin/penicillin (Corning™) and 55 μM β-mercaptoethanol (Gibco, Carlsbad, CA, USA) and stimulated with hgp100 peptide (1 μM KVPRNQDWL; Genetel Laboratories LLC, Madison, WI, USA) and recombinant human (rh) IL-2 (30 UI/mL; Miltenyi Biotec, Bisley, UK). Fresh culture medium with rhIL-2 was added on days 2 and 4. At day 6, the phenotype of the cells was analyzed by flow cytometry.

Naïve CD8^+^ T cells were purified from total splenocytes of twelve-week-old C57BL6 wild-type mice by negative selection method using the Naive CD8α^+^ T Cell Isolation Kit (MACS, Miltenyi Biotec), according to the manufacturer′s instructions. The phenotype of these cells was analyzed by flow cytometry.

### 4.4. HLA-A2-Restricted/MART-1-Specific Human CD8^+^ T-Cell Clone CdL43-1

The CdL43-1 clone was generated as described before [10]. Briefly, T cells were obtained from a fresh pulmonary metastasis of an HLA-A2^+^ melanoma patient. After mechanical tissue separation, the cell suspension was cultured at 37 °C, 5% CO_2,_ and fed every two days with RPMI 1640 medium supplemented with 10% FBS and rhIL-2 (375 UI/mL). At day 14, the CTLs were enriched using CD8 mAb-coupled beads and cloned by limiting dilution in 96-well U-shaped microtiter plates in the presence of feeders (irradiated PBMC from two allogeneic donors), plus rhIL-2 (750 UI/mL) and 12 μg/mL OKT-3 mAb. Obtained CTL clones were tested for IFN-γ production against Mel1 melanoma cell line (HLA-A2^+^, MART-1^+^), and against T2 cells pulsed with different MAA peptides. The selected clone (CdL43-1) is HLA-A2-restricted and MART-1_27–35_ specific and recognizes HLA-A2^+^/MART-1^+^ melanoma cells [10].

### 4.5. Microscopy

Confocal microscopy of fixed target tumor cell/T cell co-cultures was performed as described previously [18]. Target tumor cells were pre-loaded with 10 μg/mL AlexaFluor 647-conjugated wheat germ agglutinin (WGA; Invitrogen, Paisley, UK) according to the manufacturer′s instructions. Then, target tumor and effector cells were co-cultured for 30 min at 37 °C and 5% CO_2_ on poly-l-lysine-coated slides, at a 2:1 effector:target ratio, using 6 × 10^5^ effector cells per slide in 100 μL of complete culture medium. Cell conjugates were gently washed with PBS twice and fixed with 4% paraformaldehyde for 15 min. After gentle washing with PBS, the cells were incubated in ammonium chloride (50 mM) for 10 min. Then, the cells were permeabilized for 10 min (0.5% Triton X-100 and 0.5% FBS) and blocked with 0.5% bovine serum albumin (BSA) for 15 min. Cells were stained with a 1:500 dilution of FITC-coupled monoclonal antibody anti-Cx43 (D-7; Santa Cruz Biotechnology, Dallas, TX, USA) for 1.25 h at room temperature, and then stained with 1:1000 dilution of Hoechst 33342 (Invitrogen) and 16.5 μM rhodamine-phalloidin (R415, Invitrogen) for 15 min. Cells were analyzed with a C2+ confocal microscope (1000×, Nikon Instruments Inc., Melville, NY, USA) and a Spinning Disk Olympus BX61WI microscope (400×, Center Valley, PA, USA). The recruitment of Cx43 to the cell-to-cell contact site (cytotoxic immunological synapses) was quantified using ImageJ software (version number 1.48v, National Institutes of Health, Bethesda, MD, USA) as previously described [18].

### 4.6. Transfections

Knockdowns of Cx43 were obtained by transfection of B16F10 cells with esiRNA against Cx43 (siCx43; Mission, Sigma-Aldrich, St. Louis, MO, USA), a pool of independent target-specific siRNAs against Cx43. A siRNA of scrambled sequence (siRNA Scrambled; Ambion, Thermo Fisher Scientific, Austin, TX, USA; siScr) without target in the murine genome was used as a negative control. Transfections were performed using the Silencer siRNA Transfection Kit (Ambion, Thermo Fisher Scientific) according to the manufacturer’s instructions. Briefly, a transfection complex was generated mixing the siPORT amine transfection agent with the transfection medium OPTIMEM™ (Gibco). Then, the transfection complex was incubated for 10 min with 30 nM of siCx43 or siScr. B16F10 cells were incubated with siRNA-containing transfection complex for 6 h. After this period, the transfection complex was replaced with complete cell-culture medium. After 72 h, cells were harvested and used in different experiments.

### 4.7. Western Blot

Cells were lysed in radioimmunoprecipitation assay (RIPA) lysis buffer supplemented with 5 mM EDTA and protease/phosphatase inhibitors cocktail (all from Thermo Fisher Scientific) for 30 min on ice. Western blotting was conducted as described previously [18], loading 30 µg of total proteins per well. For protein detection anti-Cx43 (dilution 1:500; catalog no. C6219, Sigma-Aldrich), donkey anti-rabbit HRP-conjugated (dilution 1:5000; SA1-200, Thermo Fisher Scientific) and anti-β-actin IgG1 HRP-conjugated (dilution 1:10,000; SC-47778, Santa Cruz Biotechnology) antibodies were used. The membranes were revealed using the SuperSignal^®^ West Pico Chemiluminescent Substrate kit (Thermo Fisher Scientific) according to manufacturer′s instructions and analyzed in an ImageQuant LAS 500 (GE Healthcare, Uppsala, Sweden). Images were analyzed using ImageJ software (National Institutes of Health).

### 4.8. Cx43 Mimetic Peptides

Cx43 mimetic gap27 (SRPTEKTIFII) and control gap27 scramble (Scr; TFEPIRISITK) peptides were synthesized by TOCRIS Bioscience (Ellisville, MO, USA). Both mimetic and control peptides were used at 300 μM final concentration in inhibition experiments.

### 4.9. Calcein Transfer Assay

GJ-mediated cell coupling was measured using calcein transfer assay as previously described [17]. For this study, pMEL-1 CTLs, wild-type naïve CD8^+^ T cells, or human CdL43-1 CTLs were loaded with calcein-acetoxymethyl (AM) (0.7 μM; Invitrogen) for 30 min at 37 °C according to the manufacturer’s instructions. The membrane-permeable calcein-AM is hydrolyzed by intracellular nonspecific esterase, and the resulting green fluorescent hydrophilic calcein is then trapped inside the cells. B16F10 melanoma cells, MB49 urothelial carcinoma cells, and K562 lymphoma or Mel1 melanoma cells were loaded with the CellTracker Violet BMQC for 10 min (10 μM; Invitrogen), and the reaction was stopped by adding FBS for 1 minute. GJs are permeable to calcein but not to Violet BMQC. MB49 cells were pre-loaded or not with 1 μM of hgp100_25–33_ peptide (for 30 min) before co-culturing them with pMEL-1 CTLs. Violet BMQC-stained target cells were co-cultured with calcein-stained CTLs (2 × 10^5^ cells) for different times and at 1:5 or 1:3 ratios. In the case of human cells, control (Scramble; Scr) or inhibitory (gap 27) peptides were added 15 min prior to the co-cultures and incubated at 37 °C, 5% CO_2_. After co-culture, cells were collected and calcein transfer was analyzed by flow cytometry. When indicated, a cell coupling factor that takes into account the percentage of cells acquiring calcein and the level of calcein acquired by acceptor cancer cells was calculated as Cell coupling factor = (%Violet BMQC^+^calcein^+^ cells × Calcein MFI of Calcein^+^ cells)/100.

### 4.10. GrzmB Activity Assay

GrzmB activity was measured using the GranToxiLux kit (OncoImmunin Inc., Gaithersburg, MD, USA) according to the manufacturer’s protocol, and as described before [17,18]. TFL4- and NFL1-labeled B16F10, MB49, K562 or Mel1 target cells were co-cultured with pMEL-1 CTLs, wild-type naïve CD8^+^ T cells, or CdL43-1 CTLs (2 × 10^5^ cells) for 1 or 2 h at 1:5 or 1:3 ratios, in the presence of a permeable fluorogenic substrate for GrzmB. In the case of human cells, control (Scr) or inhibitory (gap 27) peptides were added to the co-culture after cell resuspension in the GrzmB substrate solution. GrzmB activity was evaluated in viable target cells (NFL1^−^TFL4^+^) by flow cytometry.

### 4.11. Flow Cytometry

Flow cytometry experiments were performed as previously described [10]. To detect Cx43, a rabbit polyclonal anti-human/mouse Cx43 antibody, directed to the C-terminal domain (C6219; Sigma-Aldrich), and a secondary donkey anti-rabbit FITC-conjugated Ab (Poly4064; BioLegend, San Diego, CA, USA) were used. The following monoclonal antibodies were used for T-cell staining: CD8α-V500 (dilution 1:500; clone 53-6.7), CD25-APC (dilution 1:1500; clone PC61), CD44-PE-Cy7 (dilution 1:500; clone IM7), Vβ13-V450 (dilution 1:400; clone MR 12.3), all from BioLegend. 7-AAD (1:1500; BioLegend) staining was used for cell dead exclusion. Samples were acquired on a FACSVerse™ (BD Biosciences, Hershey, PA, USA) and analyzed using FlowJo software (version 10.0.7; Tree Star Inc., Ashland, OR, USA).

### 4.12. ^51^Cr Release Assay

The cytotoxic activity of isolated CdL43-1 clones was measured by conventional 4-h ^51^Cr (PerkinElmer, Waltham, MA, USA) release assays using triplicate cultures in round-bottom 96-well plates in the presence of the different Cx43 mimetic peptides. Effector:target cell ratios were as indicated, on 5000 target cells/well. Specific lysis was calculated according to the following formula: percent specific lysis = ((experimental release − spontaneous release)/(maximum release − spontaneous release)) × 100.

### 4.13. Statistics

Data were analyzed using GraphPad Prism 7 (GraphPad Software Inc., San Diego, CA, USA). Statistical analyses were performed using one-way ANOVA, Tukey’s multiple comparison test, or a two-tailed Student’s *t*-test where appropriate. Differences were considered statistically significant at *p* < 0.05.

## Supplementary Materials

Supplementary materials can be found at https://www.mdpi.com/1422-0067/20/18/4509/s1.

## Abbreviations

APC	Antigen-presenting cell
Cx	Connexin
CTL	Cytotoxic T lymphocyte
NK	Natural killer
GJ	Gap junction
HLA	Human leukocyte antigen
MART-1	Melanoma-associated antigen recognized by T cells
MAA	Melanoma-associated antigen
FBS	Fetal bovine serum
BSA	Bovine serum albumin
DC	Dendritic cell
Scr	Gap27 scrambled peptide
GJIC	GJ-mediated intercellular communication
IS	Immune synapse
IFN	Interferon
GrzmB	Granzyme B
MHC	Major histocompatibility complex
TCR	T-cell receptor
IL	Interleukin
siCx43	siRNA Against Cx43
siScr	siRNA Control (scrambled)
