# Supplementary material for: Cx43-Gap Junctions Accumulate at the Cytotoxic Immunological Synapse Enabling Cytotoxic T Lymphocyte Melanoma Cell Killing
Source: Int J Mol Sci. 2019 Sep 12;20(18):4509. doi: 10.3390/ijms20184509 (PMC6769613; doi:10.3390/ijms20184509)
Supplement: Supplementary file 1 [file ijms-20-04509-s001.pdf]

Supplementary Materials:

Supplementary Figure 1. Phenotypic characterization of differentiated pMEL-1 cytotoxic T lymphocytes (CTLs) and isolated wild type *naïve* $CD8^+$ T cells.

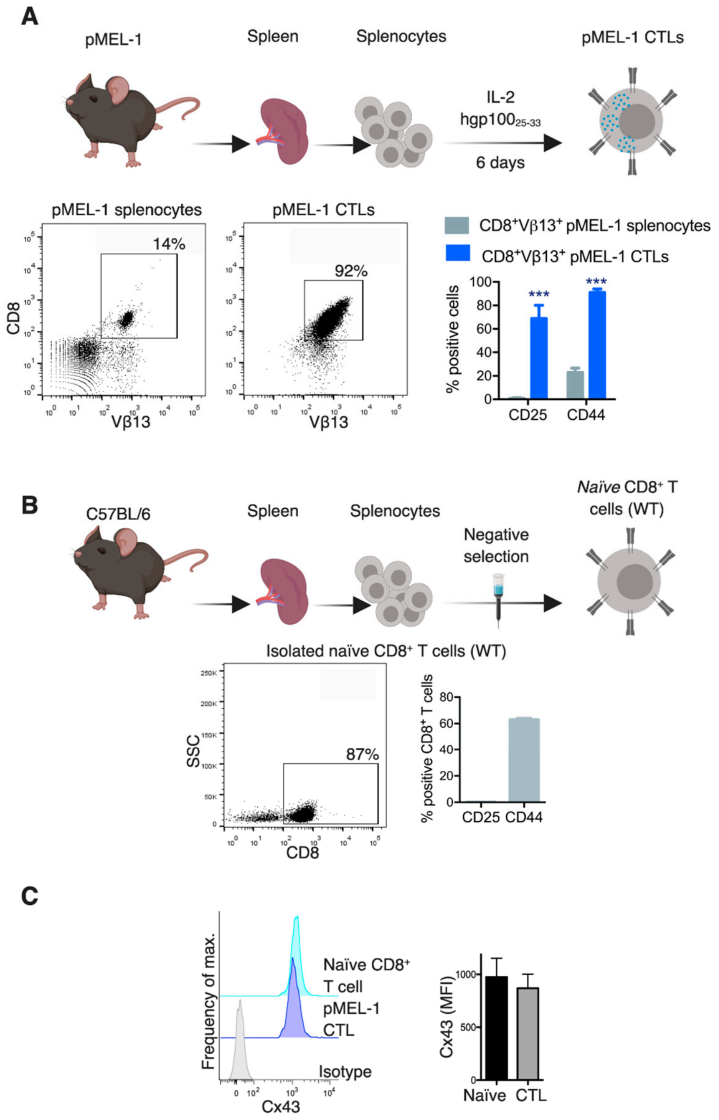

Supplementary Figure 1. Phenotypic characterization of differentiated pMEL-1 cytotoxic T lymphocytes (CTLs) and isolated wild type *naïve* $CD8^+$ T cells. A) Scheme showing the *in vitro* differentiation steps for pMEL-1 CTLs. Splenocytes isolated from pMEL-1 mice were incubated for 6 days with IL-2 and the peptide $hgp100_{25\text{-}33}$ (upper). Representative dot plots show the efficiency of pMEL-1 CTL differentiation through the expression of CD8 and the transgenic TCR chain Vβ13,

evaluated by flow cytometry in the viable ($7AAD^-$) T cell population in both pMEL-1 splenocytes and CTLs (lower, left). The expression levels of CD25 and CD44 were evaluated in the $CD8^+V\beta13^+$ T cell populations in both pMEL-1 precursor splenocytes and in *in vitro* differentiated CTLs. Bar graph shows the average expression of these markers of five independent experiments, *** $p < 0.001$, splenocytes vs CTL (lower, right). B) Scheme showing the *in vitro* isolation steps for wild type *naïve* $CD8^+$ T cells. Splenocytes isolated from C57BL/6 wild type mice were subjected to magnetic cell separation (negative selection) for *naïve* $CD8^+$ T cells (upper). Representative dot plot shows a typical isolation efficiency (87% $CD8^+$ T cells) (lower, left). The bar graph shows the percentage of isolated *naïve* $CD8^+$ T cells positive for CD25 and CD44 expression, indicating their *naïve* origin (n = 5 independent experiments) (lower, right). C) Cx43 expression was evaluated in pMEL-1 CTL and *naïve* $CD8^+$ T cells by flow cytometry using anti-CX43 specific antibodies. The bar graph shows the mean fluorescence intensity (MFI) for Cx43 in both T cell populations (n = 3 independent experiments).
